# Supplementary material for: Are carbon emissions trading and green financial instruments synergistic? -Comprehensive quantitative research based on content analysis
Source: PLoS One. 2024 Mar 7;19(3):e0298601. doi: 10.1371/journal.pone.0298601 (PMC10919601; doi:10.1371/journal.pone.0298601)
Supplement: S1 Data — (ZIP) [file pone.0298601.s001.zip › Data/地级市Z_F工作报告.zip!/╜¡╦╒/╨∞╓▌╩╨/└┤╫╘╣1⁄2╓┌║┼í╛┬φ┐╦╩2╛▌═°í┐_2008.docx]

各位代表：

　　现在，我代表市人民政府向大会作工作报告，请予审议，并请市政协各位委员和其他列席人员提出意见。

　　一、过去五年的回顾

　　2003年以来，本届政府在省委、省政府和市委的正确领导下，团结带领全市干部群众，以邓小平理论和“三个代表”重要思想为指导，深入贯彻落实科学发展观，开拓创新，拼搏进取，圆满实现了任期目标，全市经济建设和各项社会事业取得显著进步。

　　——国民经济持续健康发展，综合实力大幅提升。2007年，全市地区生产总值达到1700亿元（预计，下同），比上年增长15.2%，总量是2002年的2.27倍，五年来年均递增14.2%。财政总收入完成220.73亿元，比上年增长36%，年均递增29.6%；一般预算收入完成100.38亿元，比上年增长41.3%，年均递增28.6%。全社会固定资产投资完成960亿元，比上年增长28%，五年累计完成3143亿元，年均递增23.1%。三次产业结构由2002年的17.9:46.9:35.2调整为11.3：52.3：36.4。

　　工业经济实现新的跨越。五年累计完成工业投入1555亿元，174个亿元以上项目竣工投产；其中去年完成工业投入505亿元，组织实施亿元以上项目138项。2007年，全市规模以上工业增加值完成630亿元、比上年增长20%，占GDP比重比2002年提高12.7个百分点；规模以上工业企业达1959家，比2002年增加1216家；营业收入超亿元的企业达360家，其中超百亿元的企业4家；机械、食品、化工、建材四大支柱产业产值比“九五”末翻两番，光伏光电、煤化工、盐化工等新兴产业蓬勃兴起。城乡统筹发展步伐加快，县域经济不断提速，规模以上工业增速连续三年超过市区。在工业经济快速增长的同时，万元GDP能耗比上年下降4.5%，化学需氧量和二氧化硫排放量同比减排1.92%、14.1%。

　　新农村建设开局良好。深入落实扶持粮食、棉花生产和农机具购置补贴等支农惠农政策，全面免征农业税及附加，充分调动了农民生产经营的积极性。2007年，全市农业增加值完成192亿元，比上年增长6.5%；粮食总产达75.3亿斤，保持连续四年增产，高效农业规模化水平位居全省第一。年销售收入千万元以上的龙头企业发展到246家，农民专业合作经济组织达1896个，带动农户73万户。五年来，完成重点水利工程投资40.5亿元、农业综合开发投资11.57亿元；落实示范扶贫项目830个，受益农户达45万户；农村五件实事全面完成，新五件实事扎实推进；首批确定的150个新农村示范村有70%基本达到市级标准。

　　服务业发展态势强劲。五年累计完成服务业投入1167亿元，成功引进了金鹰国际、家乐福、沃尔玛、易初爱莲、南京中央百货、世茂置业、力宝集团、苏宁电器等大型商贸流通企业，市中心商圈日益繁荣，各专业市场和现代物流业加快发展。2007年，全市服务业增加值完成619亿元，社会消费品零售总额实现538亿元，分别比上年增长15.5%和17%。旅游经济持续快速增长，总收入五年来年均递增27.8%，两汉文化、军事文化、云龙山水等知名品牌叫响全国。金融生态环境显著改善，通讯、会展、社区服务等业态同步发展。

　　——改革开放深入推进，体制机制活力明显增强。国企改革基本完成，改制面达96%；徐钢总厂、北方氯碱、四方铝业、天能集团等21家大型骨干企业成功实现战略重组。农村各项改革扎实推进，全市农民负担比农业税改革前减轻6亿多元。市、县两级政府机构精简率分别达21.6%和16.1%，乡镇机构改革试点取得阶段性成效，行政审批事项核减幅度达72.4%。公务员制度改革、事业单位改革稳步实施，财税、金融、投融资体制改革逐步深化。民营经济快速发展，成为县域经济的重要支撑力量，私营企业、个体工商户注册资本金达到435亿元和34.5亿元，分别是2002年的4.8倍和1.6倍。

　　大招商、大开放格局逐步形成。全市上下集中力量抓招商的氛围日益浓厚，成功举办了五届徐州投资洽谈会等重大招商活动；专业招商队伍建设不断加强，产业链招商、错位招商、小分队招商、驻点招商深入开展，招商引资成功率进一步提高，2007年实际到帐注册外资4.6亿美元、增幅达88.3%，五年累计实际到帐注册外资16.1亿美元、协议利用外资42.9亿美元。成功引进了多晶硅、青岛啤酒、星丰金属、江苏金浦、中美矽晶等一批投资规模大、带动力强的重大项目；各县（市）也引进了一批大项目、好项目，铜山东亚年产150万吨钢铁、新沂迪派溢数字采油机械、邳州富山医疗、睢宁年产200万台冰柜、丰县银龙电缆、沛县丰源铝箔等项目相继开工或竣工投产。对外贸易与经济合作水平不断提高，出口总额、对外承包工程完成营业额、对外劳务合作人数年均分别递增46.5%、34.9%和34.6%。投资环境日益改善，连续四年荣登《福布斯》“中国大陆最佳商业城市”排行榜，连续两年被台湾电机电子同业公会评为“极力推荐投资城市”，2006年被评为中国投资环境百佳城市，入围中国魅力城市20强、中国城市诚信政府排行榜30强，名列跨国公司最具投资价值中国城市排行榜第六位。

　　开发区加快发展。将“松绑放权”和鼓励“自我提拔”相结合，全力支持开发区建设，开发区承载能力显著增强。五年来,徐州经济开发区和其他7家省级开发区实际到帐注册外资、自营出口、财政收入年均分别递增28.6%、85%和44.4%;累计完成基础设施投入101亿元,年均递增77.3%,形成了产业集聚、错位发展的格局，南北挂钩共建园区工作取得实质性进展。2007年，全市省级开发区实际到帐注册外资占全市总量的比重达63%，特别是徐州经济开发区强力推进“二次创业”，实现了历史性突破，实际到帐注册外资达1.8亿美元，比上年增长80%。

　　——城市化进程不断加快，城乡面貌发生巨大变化。城市规划体系逐步完善。组织完成了徐州都市圈规划、城市总体规划、分区规划、专项规划、控制性详细规划和重点地区城市设计编制工作，实现了主城区规划全覆盖，城市总体规划通过国务院审批。六县（市）总体规划、镇村布局规划、新农村示范村建设整治规划、300个三类村庄建设规划及1300个一般村庄的平面布局规划相继完成。

　　城乡建设成效显著。老城区综合改造力度不断加大，五年累计投入267亿元，实施了城市中心区综合改造、云龙湖风景区显山露水等178项城建重点工程；2007年组织实施了一批重大功能性项目，小南湖景区和汉文化景区二期工程、故黄河风光带、九里湖生态湿地公园一期工程、云龙湖秋韵园、7000亩荒山绿化等36个项目完成建设任务，和平路东延、世茂汉之源、金桥国际商务区等23个跨年度项目顺利推进。新城区建设取得阶段性成果，18条主要道路建成通车，道路绿化基本完成,行政办公中心全面竣工，综合场馆等公共配套设施项目陆续开工，呈现出整体开发的良好态势。全市大交通框架基本形成，五年完成投入121亿元，先后建成宁宿徐、京福徐州东绕城和西北绕城等高速公路，形成了“两纵两横一环”的高速公路网络，通车里程位居全省前列；近千公里的国省干线公路实现提档升级，7000公里农村公路建成使用，县镇村公路基本通达成网；京杭运河徐州段、徐洪河通航条件明显改善，港口吞吐量位居全国内河港口前列；观音机场已开通国内航线20条和香港临时包机，年旅客吞吐量达42万人次。县城镇建设累计完成投资180多亿元，全市城市化率达46.3%，比2002年提高11.1个百分点。

　　城市拆迁工作取得重大突破。大力推行阳光拆迁、和谐拆迁，实施了属地管理、定向安置、最低保障等一系列政策措施。2007年首次采取了对拆迁安置地块实行梯次转移、变动迁房为商品房、以限房价竞地价的方式建设定销商品房，并以低于市场的价格定向销售给被拆迁居民，既使被拆迁居民住上了价格便宜、地段较好的商品房，又有效平抑了房地产市场价格，加快了城市动迁和建设步伐，市区顺利完成拆迁300万平方米。

　　城市管理不断加强。“四城同创”取得重大成果，先后荣获江苏省文明城市和国家园林城市称号，国家环保模范城市创建顺利通过国家考核验收，国家卫生城市创建通过省考核验收，城市环境面貌显著改善。影响群众生活和城市发展的违法建筑拆除工作取得突破性进展，2007年共拆除违法建筑57万平方米，超过前10年总和。对城市土地全面实行了政府收储、公开招拍挂制度，进一步完善了“两级政府、三级管理、四级网络”的城市管理体制，采取了相对集中城管行政执法权与特殊领域专业管理相结合的办法，城市管理的长效机制逐步形成。

　　——社会保障体系日益健全，人民生活水平稳步提高。逐年扩大社会保障资金投入，保障范围向城镇居民、灵活就业人员、被征地农民和进城务工农民扩展，城乡低保做到了动态管理下的应保尽保，基本实现了全覆盖。2007年，市财政对社会保障领域的直接投入近9亿元，全市企业退休人员养老金月平均水平、城市和农村低保标准分别比上年提高13.8%、16.7%和14.3%；城镇职工医疗保险统筹基金支付限额由3.2万元提高到5万元，城镇居民医疗参保率达80%以上，新型农村合作医疗参合率达93.6%。就业和再就业工作力度加大，高校毕业生就业指导机制逐步完善，五年来新增转移农村劳动力77.1万人，新增城镇就业42.2万人，下岗失业人员实现就业再就业17.7万人，城镇登记失业率控制在3%以内。住房保障不断强化，2004年以来建成经济适用房40万平方米，解决了3215户困难家庭的住房问题，2007年建成廉租房100套、发放廉租房补贴443户、减免直管公有住房租金1199户。慈善事业快速发展，共募集期款2.25亿元、现款5260万元，组织实施了一系列救助项目。坚持每年为人民群众办好一批实事项目，五年累计完成实事项目58件246个子项。2007年，城市居民人均可支配收入和农民人均纯收入达到14800元、5435元，分别比2002年增长84.2%和56.3%。

　　——各项事业全面进步，社会发展日趋和谐。科教优先战略深入落实。五年来组织实施火炬计划项目187项、星火计划项目389项，2007年高新技术产业产值达160亿元、是2002年的7.1倍。12万平方米的徐州科技创业园一期工程建成使用，206家科技型企业入驻创业，中国矿业大学国家大学科技园、国家火炬计划徐州工程机械产业基地等被批准建立，11个县（市）区被认定为省级科普示范县（市）区。坚持教育优先原则，建立了义务教育经费保障机制，对义务教育阶段学校学生全部免除了学杂费，筹集助学资金6000多万元，资助家庭经济困难学生34.7万人次，减免各种费用8900多万元，为义务教育阶段学生免费提供教科书32.2万人次；累计投入30多亿元用于改善农村中小学办学条件，改造危房304万平方米，新建校舍305万平方米，全市农村中小学基本达到合格学校标准；高中阶段教育毛入学率达70%，高等教育毛入学率达37%，分别比2002年提高31.5和19个百分点；高等教育加快发展，机械技工、模特、保安等职业教育品牌逐步形成；认真落实《徐州市老年教育条例》，老年教育的规模和质量不断提高。

　　其他各项社会事业长足发展。大力弘扬新时期徐州精神，相继开展了一系列主题教育活动和群众性精神文明创建活动，市民素质和城乡文明程度进一步提高。徐州新图书馆、汉画像石艺术新馆、淮海战役纪念馆新馆、李可染艺术馆等一批文化设施建成使用，实现了每县（市）有图书馆、文化馆的目标，镇、村文化设施建设进展顺利；举办了李可染艺术节、海峡两岸楚汉文化研讨会等一系列文化艺术活动，培育形成了“书画徐州”等文化品牌。强化公共卫生体系建设，出色完成了非典、禽流感等重大传染病防治任务，食品药品监管体系逐步完善；农村改水受益人口累计达624万人、农村改厕达52万户，农村社区卫生服务机构覆盖率达91.85%，城市社区卫生服务中心覆盖率达100%。体育事业蓬勃发展，建成市体育中心等高标准体育场馆，实现了“村村有健身工程”的目标；累计承办24项次全国比赛和多项国际赛事，在第十六届省运会上取得奖牌数第三、总分第三的好成绩。人口和计划生育工作深入开展，计划生育利益导向机制不断完善，计划生育率保持在85%以上，人口出生率保持在10％。以下。平安徐州、法治徐州创建扎实推进，应急管理体系基本建立，重大信访到期结案率达98.6%。安全生产形势明显好转，事故起数和死亡人数连续五年实现“双下降”。人防、民兵、民族宗教、民政、档案、外事、侨务、台湾事务、统计、审计、物价、广播电视、无线电管理、新闻出版、气象、妇女儿童、青少年、关心下一代、老龄、残疾人、扶贫等各项事业都取得了新的进展。

　　生态环境质量不断提高。全市建成11个自然保护区和风景名胜区以及4个国家级生态示范区，自然保护区覆盖率由2002年的3.09%提高到8.74%。2007年市区绿化总量超过1300万平方米，相当于前五年增量的总和，人均公共绿地大幅增加，城市绿化覆盖率达38.81%，全市森林覆盖率达26.5%、高于全省10个百分点；市区空气质量二级以上天数由2002年的56天增加到312天；市区生活垃圾无害化处理率达85%以上，污水处理能力比2002年增长了3倍。国土资源管理工作卓有成效，保持了耕地总量动态平衡，有效发挥了保护资源、保障发展的重要作用。

　　——政府自身建设扎实开展，效率效能进一步提升。认真贯彻落实《行政许可法》，深入开展机关作风建设，群众及投资客商对机关作风的满意率大幅提高。积极推进政务公开，建立了市政府常务会议报道和新闻发言人制度，开通了市长信箱、“政风热线”和政务信息网，成立了行政效能投诉中心，市级56家具有行政处罚资格的部门和单位编制了职权目录及权力运行流程图。加快制度创新，采取了AB岗工作制、限时办结制、联审联办制、网上审批制等有效举措，在全省率先实行了规范性文件有效期制度，进一步强化了行政服务中心“一条龙”服务规范。行政监察和审计监督得到加强，党风廉政建设责任制、行政执法责任制深入落实。自觉接受市人大及其常委会的法律监督和工作监督，主动接受市政协及社会各界的民主监督，累计办理人大代表建议1629件、政协委员提案2653件，满意率均在95%以上。

　　在充分肯定成绩的同时，我们也清醒地认识到，当前经济和社会发展中还存在着不少矛盾和问题。主要是：工业结构层次偏低，经济发展方式较为粗放，完成节能减排任务的难度较大；开放型经济总体水平不高，利用外资的规模和层次亟待进一步提升；农村二、三产业发展相对较慢，基础设施条件较为薄弱，农民持续增收的难度加大，全面实现小康和新农村建设任务十分艰巨；社会保障压力加大，城乡部分群众生活还比较困难，一些事关群众切身利益的问题尚未得到根本解决；少数部门的服务意识、行政效能、创新精神与经济社会发展的要求还不相适应，政务优化、政务监督、政务廉洁等工作仍需进一步加强，投资与发展环境还要下更大的力气予以改善。对于这些问题，我们将高度重视，认真加以解决。

　　各位代表，过去的五年是全市人民思想观念和精神面貌发生深刻变化的五年，是改革开放迈出坚实步伐并取得重大成就的五年，是国民经济又好又快发展、人民群众得到实惠最多的五年，是城乡环境发生巨大变化、对外形象显著提升的五年，是各项事业协调发展、社会全面进步的五年。我们深切感到，全市经济和社会发展所取得的成绩来之不易，每一步发展、每一份成就都深深凝聚着广大干部群众的心血和汗水。五年来，全市上下坚持科学发展，把又好又快作为发展的鲜明主题；突出工业经济第一方略，把工业强市作为发展的强大引擎；强化外向带动，把招商引资作为发展的生命线；深化改革改制，把开拓创新作为发展的永恒动力；关注民生民计，把富民安民作为发展的最终目的，克服了许多矛盾和困难，积累了许多宝贵的经验，这些都将为我市今后的发展注入强大的动力。在此，我代表市人民政府，向全市广大干部群众、人民解放军驻徐部队、武警官兵和公安民警，向各民主党派、工商联、人民团体和无党派人士，向热心支持徐州现代化建设的香港特别行政区同胞、澳门特别行政区同胞、台湾同胞、海外侨胞和各界人士，向投身徐州经济社会发展的国内外投资者和企业家，表示衷心的感谢并致以崇高的敬意！

二、今后五年的展望

　　今后五年，是推进全市经济又好又快发展、全面建设小康社会、加快和谐社会建设的关键时期。我们要深入贯彻党的十七大精神，高举中国特色社会主义伟大旗帜，坚持以邓小平理论和“三个代表”重要思想为指导，全面落实科学发展观，紧紧围绕富民强市和“全面奔小康、建设新徐州”的目标，进一步解放思想，开拓奋进，全力推动各项工作取得新的更大突破。经过全市上下五年的共同奋斗，到2012年，在切实达到省定四大类18项全面小康指标要求的基础上，以更高的标准完成全面建设小康社会的新任务，把我市基本建成区域性先进制造业高地、高效规模特色农业示范基地、现代服务业之都和高品质的生态城市。

　　（一）更加重视经济发展方式的转变，形成富有竞争力的特色产业体系。着力推进产业集聚、产业转型和产业升级，围绕主导产业和支柱产业，切实强化有效投入，力争每年建设10个10亿元以上的重大工业项目，把我市基本建成全国最大的工程机械及专用车辆城、最大的光伏光电产业基地，全国重要的特色食品、新型板材家具产业基地等；加快发展现代服务业，建成省内一流、淮海经济区规模最大、层次最高、辐射带动力最强的区域性中心城市；大力发展高效规模特色农业，扎实推进新农村建设，形成城乡经济社会发展一体化格局。

　　（二）更加重视提高自主创新能力，为加快建设创新型城市奠定坚实的基础。坚持“科教兴市”、“人才强市”战略，大力促进科技成果转化和公共服务平台建设，加快创新创业人才的培养和引进，加大对自主创新的投入，力争全社会研究与开发投入占GDP的比重提高到2%以上，工业、农业科技进步贡献率分别达到50%和60%左右，基本形成布局合理、良性互动、运行高效的区域创新体系。

　　（三）更加重视生态文明建设，建立生态、节约、环保的科学发展格局。实现由“环境换取增长”向“环境优化增长”的战略转变，统筹建设市区老城区、新城区、徐州经济开发区、贾汪区、铜山新区五大组团和丰县、沛县、睢宁、邳州、新沂五大板块，构筑空间形态科学、功能结构优化、区域协调发展的城市格局，建设总量适宜、分布合理、景观优美、个性鲜明的绿化生态体系，建成国家环保模范城市、卫生城市和生态城市，形成资源节约型和环境友好型发展方式，营造优良的自然生态环境、人居环境和商务环境。

　　（四）更加重视改善民生民计，使全市人民人人享有基本保障。全面建成覆盖城乡的社会保险、社会福利和慈善事业相衔接的社会保障体系，努力使广大人民群众“就业有门路、创业有平台、物业有收入”，基本实现“学有所教、劳有所得、病有所医、老有所养、住有所居、贫有所济”的目标，切实做到人人享有基本生活保障和基本医疗卫生服务，特殊困难群体得到有效救助，消除绝对贫困现象，大多数家庭达到全面小康生活水平，群众生活质量和生活环境显著改善。

　　（五）更加重视文化建设和社会建设，开创和谐社会建设新局面。建成一大批公共文化体育设施，完善以大型公共文化设施为骨干、社区和乡镇基层文化设施为基础的文化网络，构建覆盖全市、惠及全民的公共文化服务体系，积极发展壮大文化产业，基本实现文化名市的建设目标；完善社会管理，深化各项改革，基本形成与社会主义市场经济体制相适应、有利于社会和谐的社会事业发展体制和运行机制，促进社会与经济、政治、文化建设协调发展。

三、今年政府工作任务

　　今年是深入学习贯彻党的十七大精神的关键一年，也是新一届政府任期的开局之年，做好今年各项工作意义十分重大。今年政府工作的总体要求是：以党的十七大精神统领各项工作，以邓小平理论和“三个代表”重要思想为指导，坚持好字优先、稳中求进，好中求快、优中求进，紧紧围绕转变经济发展方式和调整经济结构，着力推进改革开放和自主创新，强化招商引资的生命线地位，强力推进重大项目建设，加速新型工业化步伐，狠抓节能减排和环境保护，加快中心城市和新农村建设，切实改善民生民计，积极构建和谐社会，推动全市经济社会又好又快发展。

　　今年国民经济和社会发展的主要预期目标是：（1）地区生产总值增长14%。（2）一般预算收入增长18%。（3）全社会固定资产投资增长18%。（4）实际到帐注册外资和自营出口总额分别增长30%和20%。（5）社会消费品零售总额增长16%，居民消费价格涨幅不超过全省平均水平。（6）城市居民人均可支配收入增长11%，农民人均纯收入增长10%。（7）城镇登记失业率控制在4%以内。（8）万元GDP能耗下降5%，化学需氧量削减1.6%，二氧化硫排放量削减10.7%，环境质量综合指数达到80分以上。（9）人口自然增长率控制在6％。以内。

　　为实现上述目标，我们将扎实做好以下七个方面的工作。

　　（一）大力转变经济发展方式，加快徐州老工业基地振兴

　　坚持新型工业化第一方略，抓住省委、省政府大力支持徐州老工业基地振兴的机遇，加快推进产业集聚、产业转型和产业升级。

　　一是组织实施工业结构战略性调整。充分发挥我市重化工业优势，强化工作思路创新、技术创新、管理创新和发展路径创新，集中力量发展先进制造业，优化提升产业层次。围绕工程机械及专用车辆城建设，以徐工集团、卡特彼勒徐州有限公司等骨干企业为龙头，以徐州经济开发区、铜山经济开发区等为载体，做大做强工程机械主机、专用车辆、关键零部件和专业物流四大板块，发展壮大丰县电动三轮车产业；围绕光伏光电基地建设，打造多晶硅材料、太阳能光伏和硅半导体产业链；围绕食品产业集群建设，重点延伸发展铜山乳品饮料、睢宁特色农副产品加工等产业；围绕化工产业集群建设，加快发展沛县丰县煤化工和盐化工、新沂农用化工、邳州精细化工；围绕建材产业集群建设，不断优化邳州板材家具、贾汪水泥建材产业结构。同时，大力改造提升纺织、冶金、电力等传统产业，提高产品附加值和产业竞争力。

　　二是强化重大项目建设。今年全市计划安排重大项目140项，其中年度实施项目100项，前期推进项目40项。在工业项目建设上，突出支柱产业壮大、传统产业提升、高新产业培育等发展方向，强化规模大、质量高、效益好的项目建设，年内重点抓好江苏中能多晶硅二期和三期工程、徐工集团大型履带起重机、徐挖机械制造有限公司挖掘机扩建工程、北方氯碱一体化大化工、罗特艾德风电设备、台湾纺织工业园、徐州发电有限公司上大压小、大屯能源高精度铝板带、天虹纺织等项目建设。坚持以大项目带动有效投入，确保年内滚动实施3000万元以上工业项目600项，完成工业投入600亿元。

　　三是支持大企业大集团加快发展。充分发挥徐工集团、徐州烟厂、徐矿集团、维维集团、大屯煤电和徐州供电公司等大企业大集团的规模优势和吸纳功能，加大政策支持和引导力度，强化企业体制机制和技术创新，进一步增强核心竞争力；着力推进有条件的企业尽快上市融资，力争年内新增上市公司1家、拟上市企业2家。力争年内全市主营业务收入超亿元的企业达到400家，其中10亿元以上的企业25家。同时，抓好中小企业的扶持工作，特别是鼓励本土原创企业的发展壮大。

　　四是积极做好自主创新和节能减排工作。深入实施各类人才培养和引进工程，大力营造有利于人才辈出的政策环境和社会环境。加快国家大学科技园和创业中心服务平台建设，大力实施新一轮高新技术产业“双倍增”计划，组织实施国家和省级火炬计划项目90项，力争年内新增高新技术企业20家、高新技术产品60个。以更大的决心、更硬的措施强化节能减排工作，严格执行环保“一票否决”和节能减排目标责任制，逐步实现节能减排制度全覆盖。组织开展“百家企业节能行动”，强制淘汰规模小、资源消耗大、环境污染重的化工企业和生产线30家（条）。组织开展循环经济试点，建设一批资源节约型和环境友好型示范企业。

　　（二）大力开展新农村建设，推动农业和农村经济发展

　　坚持以农民增收为核心，以稳定粮食生产、保障粮食安全为基础，以发展现代农业、繁荣农村经济为首要任务，加快形成以工促农、以城带乡的长效机制，推进新农村建设和城乡一体化发展。一是加快发展高效规模农业。以巩固提高设施农业、特色农业发展水平为重点，突出十大优势主导产业，大力实施十六项高效农业规模化工程，在坚持依法、自愿、有偿原则的基础上，建立健全农村土地承包经营权流转有形市场，推进农村土地股份合作和适度规模经营，确保年内新增高效农业面积50万亩、新增设施农业面积10万亩以上、畜牧业规模养殖比重提高5个百分点。二是进一步提高产业化经营水平。大力发展农产品加工和营销龙头企业，力争年内直接进入农业产业化链条的农户比例达到50%；建立健全农产品现代流通体系，加大对农民专业合作经济组织的扶持力度，力争年内农产品及加工品销售收入、出口均增长15%以上。三是逐步推进城乡一体化进程。从推进城乡发展规划、基础设施、公共服务、就业社保等方面一体化入手，促进生产要素向农村流动、基础设施向农村延伸、公共服务向农村覆盖、现代文明向农村扩散。年内实现全市村庄整治规划和平面布局规划全覆盖，完成600公里的农村公路建设任务，抓好南水北调、东调南下等重点水利工程，加快农村饮水安全、河道整治和山区水源建设。把农村就业纳入全市社会就业体系，构筑被征地农民基本生活保障、农民最低生活保障、新型农村合作医疗和农村养老保险四道保障线。进一步加大对219个经济薄弱村的扶贫开发力度，确保20%的低收入农户实现脱贫目标。继续推进新农村建设，年内建成150个新农村示范村。深入实施农村新五件实事，确保完成年度目标任务。

　　（三）大力提升第三产业规模和层次，构筑现代服务业发展高地

　　重点发展生产性服务业，大力培育新兴服务业，优化提升传统服务业，力争服务业增长速度快于GDP增速，加快现代服务业之都建设步伐。一是构筑大商贸。加大中心商圈建设力度，突出抓好金鹰国际二期工程、中央国际商务广场、彭城北路步行街、苏宁综合商务广场、力宝大同街商贸开发等中心商圈重大项目建设，协调推进淮海广场、人民广场、徐州经济开发区及新城区四个市级副商圈建设，加快六县（市）商业中心建设，逐步形成“一主、四副、六中心”的发展格局。二是建设大交通。全力做好京沪高速铁路徐州段前期工作，推进高速铁路新客站建设和徐州火车站改造，开工建设丰沛铁路，积极争取开通更多的始发列车和徐连城际列车。加快徐济、新宿高速公路建设和干线公路新（改）建工程，加密与干线公路相配套的县乡公路网，实施机场路一级公路改造。抓好中运河“三改二”工程，努力提高运河通航能力。争取香港临时包机续航，继续开辟国内航线，年底力争达到25条，争取一类口岸开放补入国家“十一五”口岸发展规划。坚持公交优先发展，进一步改善城市交通和百姓出行条件。三是发展大物流。大力培育第三方物流企业，加强徐州经济开发区生产性物流基地和新城区生活性物流基地建设，突出抓好苏宁物流、南京淮海医药物流、交广物流、新长江物流、宝通物流、淮海经济区农副产品物流中心等重大物流项目，加快形成区域性物流品牌和大物流发展格局。四是繁荣大市场。围绕提高市场集聚度和发展层次，引导汽车及配件、建材装饰、食品蔬菜向城区东三环市场带集聚，煤炭、钢材、五金、家具向沿运河市场带集聚，加快汽配城和工程机械展示中心建设，推进淮海蔬菜批发市场与黄淮农副产品批发中心的整合提升。注重发挥九里区土地资源丰富、交通便利等优势，积极培育和建设大市场。继续加强县级区域性特色市场建设，完善地域特色鲜明的市场体系。五是振兴大旅游。发挥两汉文化、军事文化和山水文化等知名品牌优势，抓好徐州汉文化景区、云龙湖风景区、马陵山景区等景区景点建设，做好吕梁风景区规划和启动工作，打造点线面相结合的旅游精品。全面发展金融、信息、科技、会展和社区服务业，形成多业并进的发展态势。

　　（四）大力推进招商引资，不断增强发展后劲

　　坚持外资、民资、国资三资并举，三次产业齐头并进，大中小项目整体联动，推进招商引资向招商选资转变。

　　一是着力提升招商引资的质量和效益。把主攻目标放在科技含量高、产业带动力强、产出效益好、就业容量大的项目上，强力推动产业结构调整、土地集约利用、生态环境保护和社会就业扩大。突出产业链招商，力争在引进工程机械发动机、液压件和重卡项目上取得重要突破，在引进光伏光电上下游产品和生物医药、电子设备等高新产业项目上实现重大进展。切实强化专业招商队伍建设，进一步提高招商人员的业务水平和实战技能。突出重点地区招商，努力使我市成为日韩资本南下和港台资本北上的重要承接地。在扩大制造业利用外资的同时，吸引更多的国内外资金、技术和人才流向高效规模特色农业、现代服务业、文化产业和各项社会事业。

　　二是不断加强开发区载体建设。按照城市总体规划、土地利用规划和新农村建设规划要求，推动徐州经济开发区和其他7家省级开发区积极开展土地整理，合理提高项目投资强度、建筑密度，推行多层厂房建设，促进土地集约利用。进一步完善产业发展规划，强化基础设施配套和服务平台对接，加强专业片区和特色园区建设，深化南北开发区挂钩合作。放手放权鼓励开发区深化管理体制改革，努力形成产业集聚度高、质量效益好、国际竞争力强的多功能综合性示范区。把徐州经济开发区作为重中之重来抓，努力推进其晋升为国家级开发区。

　　三是继续打造优良投资环境。从解决客商最关切、最现实的问题入手，制定出台缓解资金和土地等要素制约的政策措施，有效控制商务成本过快增长。进一步简化外资审批程序，确保职权下发到位、手续简化到位、全程服务到位，营造“绿卡保障、特事特办”和“阳光服务、一条龙服务”的投资环境。

　　（五）大力提高中心城市承载力和辐射力，积极推进徐连一体化

　　加快中心城市建设，着力打造现代化特大城市和徐州都市圈核心城市。

　　一是完善城乡发展规划。全面实施城市总体规划，深化细化城市建设规划、控制性详细规划和各项专业规划，继续加快村庄规划编制工作。严格落实城市违法建筑“谁主张、谁负责”的责任机制，强化规划公示和公众参与制度，形成自觉遵守城市规划、严格执行城市规划、主动监督城市规划的制度约束。

　　二是实施一批精品工程。突出抓好规划设计、拆迁保障和施工管理等关键环节，以“一区一圈、两湖两山、三房四河、五路六园 ”建设为重点，大力实施“新城建设、山水亮丽、环境提升、公共文化、道路畅通和商贸繁荣”六大类工程。在老城区建设中，重点推进市民广场建设、九里湖生态湿地公园续建、和平路东延沿线增绿等山水亮丽工程；大力实施旧村改造、77个老小区整治，淮海路、徐丰路和徐沛路沿线综合整治等环境提升工程；切实抓好地方档案馆和历史馆、音乐厅、美术馆、新闻文化中心、广电中心、科技馆、民间博物馆区和汉文化演艺中心等公共文化工程；加快实施西安路贯通、天齐路南延、黄河北路西延等道路畅通工程；全力推进中央国际商务广场等商贸繁荣工程。在新城区建设中，重点抓好基础配套设施完善和景观绿化提升等工程，新建13条道路和11座桥梁，加强生态绿化建设，启动综合医院、学校等公共配套设施建设，加快规划馆、行政服务中心等公共场馆建设，有序推进定销商品房开发以及综合商住项目建设。

　　三是提高城市经营管理水平。着力抓好城市品牌、产业和土地经营，推进城市管理由粗放型向精细化转变、随意性向规范化转变、计划调控向市场化转变。不断强化城区政府及街道办事处的城市管理职能，扎实推进“城管进社区”工作；全面实施城市绿化、保洁等领域的招投标制度，深入开展道路市容环境、老旧小区、户外广告、集贸市场等专项整治。以政府投资为引导，构建项目开发建设平台，加快推行委托代建制，进一步拓宽城市建设与发展的投融资渠道。

　　四是加强县城镇建设。着眼于沿东陇海线产业带城市群的迅速崛起，加强对县城镇建设的政策扶持和业务指导，进一步强化丰县、沛县、睢宁、邳州、新沂等五个副中心城市功能，择优培育10个重点中心镇，逐步形成布局合理、结构有序的城镇发展体系。

　　（六）大力深化改革开放，增强促进发展的新动力

　　把改革开放作为挖潜创新的强大动力，进一步推动体制机制转变和优化。一是抓好国企改革扫尾工作。全面完成市属国企改革改制，切实解决国企改制历史遗留问题；整合改制后资产空壳化企业，组建新的资产经营公司。进一步完善改制企业法人治理结构，加强国有资产监管，开展外派董事试点工作，着力推进企业体制机制创新。二是有序推进政府机构和事业单位改革。按照上级部署稳步实施政府机构改革，进一步转变政府职能。深化事业单位改革，完善符合事业单位特点的社会保障举措和人员分流安置办法。适时全面推开乡镇综合机构改革。三是稳步实施财政金融等项改革。围绕完善公共财政体系，着眼于财力与事权相匹配，强化非税收入收缴管理和部门预算制度建设，提高公共服务能力和水平。继续推进商业银行股份制改革和农信社改革，吸引国内外银行及保险机构来我市设立分支机构。四是着力壮大民营经济实力。完善鼓励扶持民营经济发展的配套措施，加快推进民营企业担保公司和担保协会建设，培育发展新型农村合作金融组织，开展只贷不存的民间合作金融组织试点，加大对原创型中小民营企业的贷款扶持力度，切实解决民营企业贷款难问题。五是进一步提升经济国际化水平。转变外贸增长方式，优化出口商品结构和市场结构，促进加工贸易转型升级，扩大精深加工产品出口。支持有条件的企业特别是民营企业率先“走出去”，进一步拓展建筑、煤矿、机械、网架、公路工程等领域的合资合作，开辟国际经济合作新格局。

　　（七）大力开展“三业六有”工程建设，加快构建和谐社会

　　以“三业六有”工程建设为主线，完善政策，健全制度，强化措施，着力增强改善民生的普惠性和经济社会发展的协调性。

　　一是切实解决关系民生民计的重大问题。坚持以鼓励创业扩大就业、增加物业，充分发挥社保补贴、小额担保贷款、税费减免、职业技能培训补贴在促进群众创业中的作用，建立创业风险担保基金，不断拓宽广大群众增加物业性收入的渠道；强化对就业困难人员、“零就业”家庭、大中专毕业生和农村转移劳动力的就业帮扶，确保全年城镇新增就业7.5万人、城镇失业人员就业再就业3.5万人、农村劳动力转移就业9万人。着眼实现“学有所教”，取消义务教育阶段学校有关服务性收费项目，继续对义务教育阶段家庭经济困难学生实施“一免一补”，加大对城市低保家庭子女完成学业的资助力度，扩大残疾儿童接受基础教育的机会。着眼实现“劳有所得”，完善就业市场服务体系和人才资源体系，认真落实劳动合同、工资集体协商、最低工资标准和工资支付制度，努力让有劳动能力和就业愿望的各类劳动者得到有效的就业机会，依法合理地获取劳动所得。着眼实现“病有所医”，加快推进城镇居民医疗保险制度，力争年内覆盖率达到90%以上；扎实推进“农民健康工程”，新型农村合作医疗人均筹资标准提高到100 元、参合率达95%；建立和完善城乡医疗救助制度，强化药品市场监管，改善农民工就医条件，扶持“爱心医院”建设发展。着眼实现“老有所养”，扩大企业职工基本养老保险覆盖面，提高企业退休人员养老金保障水平并确保按时足额发放，完善新型农村养老保险政策体系，对特殊困难的老年人给于低保补助；加快城乡养老设施建设，建立以居家养老为基础、社区养老为依托、机构养老为补充的社会化养老服务体系。着眼实现“住有所居”，年内开工建设50万平方米的经济适用房和120万平方米的限价定销商品房，积极探索政府回购“二手房”等创新模式，力争用三年时间基本解决全市住房困难低收入群体的住房难题，努力使住房困难的低保家庭住得上廉租房、低收入家庭买得起经济适用房、新就业人员租得起房；力争用两年时间基本完成全市老旧小区改造任务。着眼实现“贫有所济”，年内募集4000万元以上慈善资金，并通过三年的积累，建立现款1.5亿元规模的慈善基金，形成滚动发展的良性机制，不断强化对弱势群体的慈善救助。继续为人民群众办好9类实事工程。

　　二是统筹发展各项社会事业。全面贯彻党的教育方针，继续推进教育均衡化发展和教育现代化进程，加快学校布局调整和资源整合，强化对薄弱学校的投入，力争用三年时间基本实现全市教育现代化目标，不断满足群众对优质教育的需求；加快高等教育资源整合步伐，抓好徐州工程学院新校区建设，推进职业教育提档升级，促进民办教育健康有序发展，继续办好各类老年大学。推进文化名市建设，以发展公益性文化为重点，加快文化设施建设，培育形成一批有较强影响力的文化产业集团，推动文化人才队伍更加壮大。实施医药卫生体制改革，加快建立比较健全的基本医疗卫生制度，建立覆盖城乡居民的公共卫生服务体系、医疗服务体系和药品供应保障体系。大力开展“全民健身与奥运同行”活动，积极推进城市社区和乡镇体育设施建设，开工建设新体育场。加快人口与计划生育公共管理和服务平台建设，切实稳定低生育水平，综合治理出生人口性别比偏高问题，提高出生人口素质。深入开展“放心消费”创建活动，坚持不懈地抓好产品质量和食品药品安全，强化物价监控，提高群众的消费满意度。加强行业拥军、科技拥军，提高拥军优属整体水平。进一步抓好人防、民兵、民族宗教、档案、外事、侨务、台湾事务、统计、广播电视、无线电管理、新闻出版、气象、妇女儿童、青少年、关心下一代、老龄、残疾人等方面的工作，促进社会各项事业协调发展。

　　三是加快生态环境建设。下大力气做好环境保护敏感区、生态保护核心区的环境治理工作，强化小沿河水源地保护，实施供水管网改造工程，确保饮用水安全。加大故黄河下游段及奎河水质综合治理力度，积极实施区域尾水导流工程，建设11座污水处理厂。加快生活垃圾焚烧发电厂建设，提升垃圾资源化、无害化利用水平。大力实施“绿色生态徐州”建设，年内完成四环路以内8000亩山地的绿化任务，市区新增绿地1330公顷，全市完成造林绿化17万亩，力争城市绿化覆盖率达40%以上、全市森林覆盖率提高到27.5%。

　　四是全力维护社会稳定。紧紧围绕新一轮平安徐州、法治徐州建设目标，全面建立布局合理、内外结合、资源共享、管理规范的社会治安防控网络，健全突发公共事件应急管理体制和运行机制。坚持安全发展，强化安全生产监管，防止重特大事故发生。完善社会矛盾纠纷大调解机制，健全法律援助体系，全面完成“阳光信访”工程建设，进一步畅通人民群众合理诉求渠道，增强基层控制、化解信访矛盾的能力，加大平安创建工作力度，确保进入江苏省“社会治安安全市”行列，人民群众对社会治安的满意度不断提高。

　　四、加强政府自身建设

　　面对新的形势和任务，我们将进一步强化政府自身建设，教育广大机关工作人员，深刻领会科学发展观的内涵，提升落实科学发展观的能力，全力推动政府机关作风明显改善、效率效能明显提升、服务意识明显增强、依法行政水平明显提高，加快形成科学发展的格局。

　　（一）不断深化政务公开，全面提高政府工作透明度。突出“服务事项、服务承诺、收费依据、办事时限、责任追究”等关键环节，进一步扩大各类行政管理和公共服务事项的公开范围，尽快实现全市各类行政职权的全过程动态公开和全流程接受监督。对涉及专业性、技术性较强的重大事项，建立健全专家论证、技术咨询和决策评估机制；对涉及群众切身利益的重大决策，建立健全公示、听证制度；对涉及财权、人权、社会资源分配权等重要职权的行使，主动接受质询和监督。完善主动公开和依申请公开等配套制度，推动政务公开深入开展。

　　（二）继续推进政务优化，强力推动政府工作提速增效。全面推进政企分开、政资分开、政事分开、政府与市场中介组织分开，更多地运用市场化的思路和办法配置要素资源，健全市场监管体系。继续深化行政审批制度改革，进一步清理、削减、合并审批事项，压缩审批办理时限，提高集中审批办结率。大力提高市行政服务中心事项入驻率和窗口直接办结率；促进服务职能重心下移，在全市114个镇加快建立便民服务中心。扎实推进精文简会，不断增强工作的计划性，采取重大信息直报、办文情况网上公开、延时催办督办等方式提高办文办事效率。推动各级各部门强化服务意识，把岗位责任制、AB岗工作制、服务承诺制等制度贯穿于政府工作的各个层面，使广大公务员加快形成优良的服务习惯。加快电子政务建设，逐步实现行政许可、行政审批和服务事项的网上申报、网上办理，推动政府效能建设迈上新台阶。

　　（三）强化政务监督考核，切实增强政府执行力。坚持履职与问责的有机结合，充分发挥监察、审计等行政专门监督机关作用和各地各部门的内部监督职能，建立健全失职必罚、渎职必究的责任关联与利益导向机制，严肃查处有令不行、有禁不止和不作为、乱作为等行为，以更大的力度克服“中梗阻”、打破“潜规则”，努力消除无所作为的“第三种状态”。严格执行政务绩效考评制度，强化对行政机关运用权力、履行职责、推动工作落实的考核管理和激励约束。主动接受市人大及其常委会的法律监督、工作监督，市政协的民主监督以及新闻舆论监督和群众监督，加强政府部门与民主党派的对口联系，积极探索和完善政务监督员制度，促进依法行政、科学施政。

　　（四）严肃抓好政务廉洁，牢固树立清正廉明的政府形象。教育广大公务员务必继续保持谦虚、谨慎、不骄不躁的作风，务必继续保持艰苦奋斗的作风。全面落实党风廉政建设责任制，坚持标本兼治、综合治理、惩防并举、注重预防的方针，更加注重治本，更加注重预防，更加注重制度建设，加快建立健全教育、制度、监督并重的惩治和预防腐败体系。着力纠正损害群众利益的突出问题，有效规范各类学校收费标准，继续纠正医药购销和医疗服务中的不正之风。严格执行经营性土地招拍挂出让制度、国有产权交易监管制度、工程建设招投标制度、专项基金使用管理制度和政府采购制度等，真正做到用制度管权、按制度办事、靠制度管人，全力打造勤政为民、廉洁从政、务实高效的公务员队伍。

　　各位代表，新的时期孕育新的机遇，新的征程充满新的挑战。让我们在党的十七大精神指引下，在省委、省政府和市委的正确领导下，聚精会神搞建设，一心一意谋发展，以更加昂扬的斗志、更加扎实的工作，开拓进取、顽强拼搏，为早日实现全面建成小康社会的目标而努力奋斗！
